# Surface Accumulation of Cerium, Self-Assembling Peptide, and Fluoride on Sound Bovine Enamel

**DOI:** 10.3390/bioengineering9120760

**Published:** 2022-12-03

**Authors:** Konstantin Johannes Scholz, Karl-Anton Hiller, Helga Ebensberger, Gerlinde Ferstl, Florian Pielnhofer, Tobias T. Tauböck, Klaus Becker, Wolfgang Buchalla

**Affiliations:** 1 Department of Conservative Dentistry and Periodontology, University Hospital Regensburg, Franz-Josef-Strauß-Allee 11, 93053 Regensburg, Germany; 2 Institute of Inorganic Chemistry, University of Regensburg, Universitätsstr. 31, 93047 Regensburg, Germany; 3 Department of Conservative and Preventive Dentistry, Center for Dental Medicine, University of Zurich, Plattenstrasse 11, 8032 Zurich, Switzerland

**Keywords:** chemical composition, EDX, caries prevention

## Abstract

The accumulation of caries-preventive compounds on sound enamel is crucial in order to improve the inhibition of carious lesion initiation. The aim of this research was to investigate the initial accumulation of cerium, oligopeptide p11-4, and fluoride from NaF or amine fluoride (AmF) on sound enamel in vitro by means of energy dispersive X-ray spectroscopy (EDX). Polished bovine enamel specimens (n = 120 from 60 teeth) were fabricated. Out of these, 12 specimens each were treated with CeCl_3_ (cerium(III) chloride heptahydrate 25%), oligopeptide p11-4 (Curodont Repair, Credentis), NaF (10,000 ppm F^−^), AmF (amine fluoride, Elmex Fluid, CP-GABA GmbH, 10,000 ppm F^−^), or Aqua demin (control). After rinsing with water, the surface elemental composition (Ce, N, F, Ca, P, O, Na, Mg) was measured (EDX; EDAX Octane Elect detector, APEX v2.0), expressed in atomic percent (At%) and analyzed (non-parametric statistics, α = 0.05, error rates method). Another 12 specimens per treatment group were fabricated and used for analyzing accumulation in cross-sections with EDX linescans and two-dimensional EDX-mappings. The surface median atomic percent of cerium (At%Ce) was 0.8 for CeCl_3_, but no Ce was found for any other group. N, specifically for oligopeptide p11-4, could not be detected. Fluorine could only be detected on fluoridated surfaces. The median atomic percent of fluorine (At%F) was 15.2 for NaF and 17.0 for AmF. The Ca/P ratio increased significantly compared to the control following the application of NaF and AmF (*p* < 0.001), but decreased significantly for CeCl_3_ (*p* < 0.001). In cross-sectioned specimens of the CeCl_3_-group, 12.5% of the linescans revealed cerium at the enamel surface, whereas 83.3% of the NaF linescans and 95.8% of the AmF linescans revealed fluorine at the enamel surface. Following the application of oligopeptide p11-4, no traces of N were detectable. In the depth of the samples, no signal was detected for any of the corresponding elements exceeding the background noise. Cerium and fluorine (from both NaF and AmF), but not the oligopeptide p11-4, precipitated on sound enamel.

## 1. Introduction

Untreated caries of permanent teeth is a prevalent condition with an estimated 2 billion worldwide cases in 2019 [1]. Coronal carious lesions typically start with lesion initiation in sound enamel [2]. Hence, the approach to combat caries is twofold: to prevent demineralization of sound enamel and to promote the remineralization of previously demineralized areas [3,4]. For all cariostatic compounds that act in addition to the local presence of ionic calcium and phosphate, adherence or accumulation at the enamel surface is therefore a preferred quality in order to positively influence the balance between demineralization and remineralization during daily acidic challenges [5].

Fluorides, e.g., applied in form of dentifrices or varnishes, have been used for a long time in the prevention and treatment of initial carious lesions [6,7]. These act primarily via local effects, for example through the initial formation of calcium fluoride (CaF_2_) on the enamel surface at acidic pH [8,9,10] or through accumulation within the hydration layer of enamel crystals and diffusion of 1–2 nm into these crystals [11]. In addition to fluoride, for which cariostatic effects are well documented, a number of novel, potentially cariostatic compounds have emerged.

Lanthanoid compounds such as cerium salts, although not yet used clinically, have recently shown anti-erosive and cariostatic potential [12,13,14]. Although not fully understood, the incorporation of cerium at the positions of calcium in the crystal lattice of hydroxyapatite may increase acid resistance. The lower solubility of cerium phosphate or cerium-substituted apatite, compared to corresponding naturally occurring calcium phosphates, may explain a potential caries-preventing effect of cerium in enamel [15]. The oligopeptide p11-4 (C_72_H_98_N_20_O_22_), designated as a *self-assembling peptide*, is described to bind calcium ions [16,17]. Nevertheless, oligopeptide p11-4 also may inhibit initiation of initial enamel caries by binding to sound enamel, due to its calcium-binding capacity [17].

In view of its potential properties to prevent enamel demineralization, it was the aim of this study to investigate the initial surface accumulation of cerium and oligopeptide p11-4 on sound enamel in vitro and compare these with fluoride from NaF or amine fluoride, both well-established compounds in caries prevention. The corresponding null-hypothesis of the study was that topical application of cariostatic agents based on cerium(III)-chloride, oligopeptide p11-4, amine fluoride, or sodium fluoride has no significant influence on the elemental composition of sound enamel.

## 2. Materials and Methods

For all in vitro experiments, permanent inferior incisors of freshly slaughtered bovine animals were extracted and stored at 4 °C for a maximum of 3 months in 0.5% chloramine solution before use.

### 2.1. Enamel Preparation and Treatment

**Figure 1:**
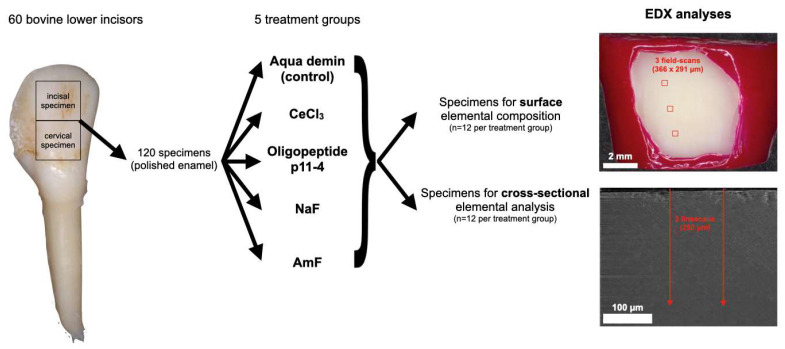
Flowchart of the experimental steps. From 60 bovine lower incisors, 120 polished enamel specimens were randomly allocated to 5 treatment groups: Aqua demin (control), cerium(III)-chloride (CeCl_3_), oligopeptide p11-4, sodium fluoride (NaF), and amine fluoride (AmF). The surface elemental composition (Ce, N, F, Ca, P, O, Na, Mg) was measured in three fields of the treated enamel surface using energy dispersive X-ray spectroscopy (EDX). The accumulation on the surface was analyzed in cross-sections by EDX linescans and two-dimensional EDX element mappings.

The crowns from 60 bovine inferior incisors were hand-sectioned into 120 equally-sized labial enamel specimens with underlying dentin (10 × 10 × 3 mm; one incisal and one cervical specimen per tooth) using a cutting disc (Superdiaflex H 365F 190 Horico Dental, Berlin, Germany) under copious water cooling. Roots and pulpal tissue were removed. The central enamel region of each specimen was ground flat and polished under continuous water cooling with Si-Carbide paper (Metaserv Motopol 8, Buehler, Leinfelden-Echterdingen, Germany; 150 rpm, FEPA P1200, CarbiMet, 40 s, FEPA P4000, MicroCut, 60 s; both Buehler, Germany). After polishing, each specimen consisted of at least a layer of 1 mm of enamel and 1 mm of underlying dentin. The dentin on the pulpal side of the specimen and the unpolished marginal enamel areas were covered with nail varnish. The resulting 120 specimens (Figure 1) were randomly allocated to 5 treatment groups (n = 24): CeCl_3_ (25% cerium(III)chloride heptahydrate in aqueous buffer-solution, pH = 4; Merck KGaA, Darmstadt, Germany), oligopeptide p11-4 (pH = 6.2; Curodont Repair, Credentis, Windisch, Switzerland), NaF (sodium fluoride, 10,000 ppm F^−^ in aqueous buffer-solution, pH = 4; Merck KgaA, Darmstadt, Germany), AmF (amine fluoride, 10,000 ppm F^−^, pH = 4; Elmex Fluid, CP GABA GmbH, Hamburg, Germany), and Aqua demin (control). The solutions according to treatment groups were applied on the polished sound enamel. For CeCl_3_, NaF and AmF, 0.1 mL of the solution was passively applied from a pipette, left for 60 s, and subsequently rinsed off for 30 s using demineralized water. For oligopeptide p11-4, one unit of approximately 60 µL of the solution was applied using the applicator from the Curodont system, left for 300 s, and subsequently rinsed off for 30 s using demineralized water. For Aqua demin (control), 30 s rinsing with demineralized water (1.82 × 107 µSv; TKA GenPure, TKA xCAD, TKA Wasseraufbereitungssysteme GmbH, Niederelbert, Germany) without further treatment was performed.

### 2.2. Surface Visualization (LV-SEM)

All 12 specimens from every group were dried in an exsiccator using activated silica gel (Silica-Gel with indicator Orange-Gel, Merck, Germany) for 24 h. Within 4 h they were mounted onto aluminum stubs (Baltic Präparation, e.K., Wetter, Germany) using double-sided adhesive carbon discs and conductive adhesive paste (Leit-Tab and Leit-C-Plast, Baltic Präparation e.K.). Exemplary superficial SEM micrographs (FEI Quanta 400 FEG, Thermo Fisher Scientific, FEI Deutschland GmbH, Dreieich, Germany) were taken from the enamel surface in low vacuum mode (secondary electron mode, 1.5 Torr, accelerating voltage 10 kV, working distance 10 mm, horizontal field width 10.82 µm) without previous sputtering, using a large field detector (LFD) and pressure limiting aperture (PLA).

### 2.3. Surface Elemental Composition (EDX)

Using the same specimens, the surface elemental composition was measured in three fields (366 × 291 µm) at a distance of at least 500 µm from each other and at least 500 µm within the margin of the treated enamel surface (Figure 1), using EDX (EDAX Octane Elect detector, APEX v2.0, AMETEK EDAX GmbH, Weiterstadt, Germany) and calibration with standard customized coefficients (SCC). The EDX measurements were performed in low vacuum mode (PLA, 1.5 Torr, accelerating voltage 10 kV, working distance 10 mm, 50 µm aperture, 100 live seconds, amp time 3.84 µs, image resolution 1024 × 800 pixels). Atomic percent (At%) of the elements Ce, N, F, Ca, P, O, Na and Mg were calculated from every field. At%Ce, At%N, or At%F were the target parameters of the experiment being selective indicators for precipitation of CeCl_3_, oligopeptide p11-4, or fluorides. C, Cl, and Si were not included in the analysis, because they were not within the scope of the study, and their concentration may be influenced by storage and polishing procedures.

### 2.4. Cross-sectional Elemental Analysis (EDX)

From the remaining 60 specimens (12 per group), cross sections through the specimen perpendicular to the enamel surface were made following treatment to analyze surface accumulation “from the side” and potential in-depth penetration (Figure 1). Before cutting, the enamel surface was gently air-dried, covered with Clearfil SE Bond (Kuraray, Chiyoda, Japan) and low viscosity bulk-fill composite (SDR flow+, Dentsply Sirona, York, NY, USA), and light cured according to the manufacturer instructions (VALO curing light, up 5919-I, Ultradent Products, South Jordan, UT, USA) in order to mechanically stabilize the enamel surface during the following cutting procedure. Subsequently, the specimens were centrally cut perpendicular to the enamel surface using a saw microtome (Leitz 1600; Leica Microsystems, Wetzlar, Germany), dried and mounted as described for surface elemental composition specimens.

In order to verify the accumulation of the target elements Ce, N and F on the enamel surface, cross-sectional EDX linescans were taken perpendicular from the cut enamel surface, including the surface and deeper areas (FEI Quanta 400 FEG, EDAX Octane Elect detector, APEX v2.0), using low vacuum mode (1.5 Torr, PLA, accelerating voltage 10 kV, working distance 10 mm, aperture 50 µm). Within each scan, the counts of either Ce, N, or F were used as target elements. For each specimen, two parallel central EDX linescans with a distance of 100 µm from each other and perpendicular to the original enamel surface were recorded. Each line comprised a row of single measurements with 2 µm line width along a length of 250 µm with 0.9 µm intervals in between two single measurements (dwell time 20 ms; amplification time 7.68 µs; 50–80 frame iterations to collect necessary counts). For each element, the EDX counts were plotted against the sample depth, and a two-dimensional fit (TableCurve 2D, v5.01 S4STAT, Chicago, IL, USA) was applied. For every linescan, the counts of the target element in >100 µm depth from the enamel surface resembled a horizontal line and were therefore defined as background noise. From all fitted EDX linescans, the functions obtained by TableCurve 2D that showed a peak of EDX counts of Ce, N, or F at the enamel surface was counted and evaluated as an additional indicator of a consistent accumulation of the target elements.

### 2.5. Determination of Nitrogen in the Oligopeptide p11-4 Delivery-System (Curodont Repair)

No information on the concentration of oligopeptide p11-4 within Curodont Repair is provided by the manufacturer. Therefore, and because of the results of the above described experiments, the nitrogen content of Curodont Repair was determined by EDX. Hitherto the relative elemental composition of the manufacturer’s applicator brush alone, the oligopeptide p11-4 fluid alone and oligopeptide p11-4 fluid applied according to manufacturer’s instructions using the respective applicator brush were analyzed. The fluids were applied on aluminum stubs that were covered with adhesive carbon discs (Leit-Tab) and allowed to dry without rinsing off. The residue was analyzed for its elemental composition with respect to C, N, O, Na, and S using EDX (all measurements: low vacuum mode, 1.5 Torr, accelerating voltage 10 kV, horizontal field width 340 µm). Additionally, the Leit-Tab without test materials was examined.

### 2.6. Data Analysis

For surface EDX data, non-parametric statistical procedures were used to analyze At% of respective elements (SPSS version 25.0, IBM, Armonk, USA). The median of the three measured fields per specimen was used as the representative value of every specimen. Ca/P ratios were calculated. For all groups, medians and 25% and 75% percentiles were calculated from the specimens’ representative values. The Mann–Whitney U Test was used to test for statistically significant differences between groups. The level of significance was set to ∝  =  0.05 and adjusted to $\propto^{*}\left(k\right)=1-\sqrt[{k}]{1-\propto }$ with *k* = number of pairwise tests to be considered in case of multiple comparisons according to the error rate method [18].

## 3. Results

**Figure 2:**
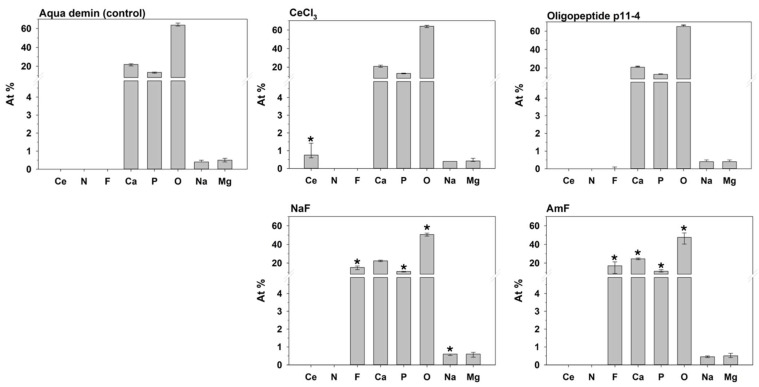
Median and 25–75% percentiles of the elemental composition [At%] at the enamel surface displayed in a separate diagram for each group (n = 12). For a single element, an asterisk indicates a significant difference between the respective group and the control (Aqua demin).

**Figure 3:**
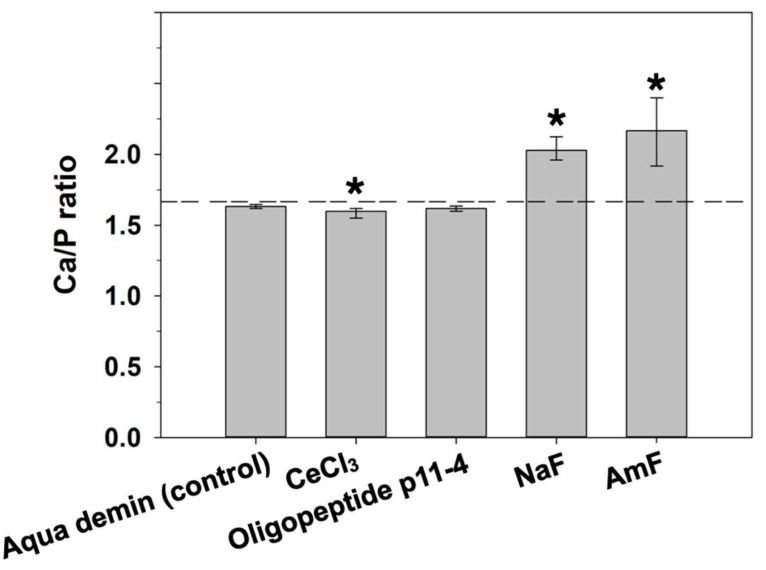
Ca/P ratio of the respective groups (median and 25–75% percentiles; n = 12) at the enamel surface. Asterisks indicate significant differences compared to the control (Aqua demin). Dashed line shows stoichiometric ratio (1.667) of hydroxyapatite.

Relative atomic percent [At%] composition at the enamel surface and the Ca/P ratio of the investigated groups are shown in Table 1 and Figure 2 and Figure 3.

Control enamel surfaces (Aqua demin, treatment with deionized water only) comprised Ca, P, O, and, to a lesser degree, Na and Mg. The Ca/P ratio in the control group was near the ratio of stoichiometric hydroxyapatite of 10:6 = 1.667. None of the target elements Ce, N, or F were found. Cerium was found on enamel surfaces after cerium(III)-chloride application (group CeCl_3_) only. Nitrogen could neither be detected after application of oligopeptide p11-4 that contains nitrogen, nor in any other group. Fluorine was always found after application of NaF or AmF, but not in any other group. According to the error rate method (k = 8), CeCl_3_, NaF, and AmF differed significantly from control (Aqua demin) regarding their elemental compositions, but oligopeptide p11-4 did not.

In detail, NaF application also led to significantly more At%Na (*p* ≤ 0.001) compared to Aqua demin, but less At%O and At%P (*p* ≤ 0.001). Treatment with amine fluoride (AmF) also exhibited significantly more At%Ca (*p* ≤ 0.001) compared to Aqua demin, but significantly less At%O (*p* ≤ 0.001), and At%P (*P* = 0.003). A significant increase of the Ca/P ratio (Figure 3) compared to the control treatment (Aqua demin) was found for NaF (*p* ≤ 0.001) and AmF (*p* ≤ 0.001), in contrast to CeCl_3_, which showed significant decreased Ca/P ratio (*p* ≤ 0.001) compared to the control treatment (Aqua demin). Oligopeptide p11-4 did not show a difference compared to control (Aqua demin) in the Ca/P ratio. Furthermore, regarding the both fluorine-containing test materials, application of NaF led to significantly more At%Na (*p* ≤ 0.001) and significantly less At%Ca (*p* ≤ 0.001) as compared to application of AmF, but there were no significant differences in At%F.

**Figure 4:**
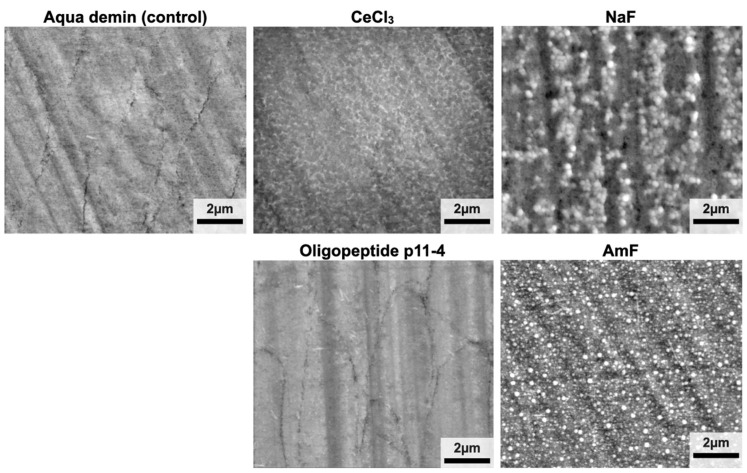
Low vacuum (secondary electron mode) SEM images. Globular precipitates comparable with CaF_2_ deposits were visible for groups NaF and AmF. The surfaces of specimens from group CeCl_3_ showed net-like superficial deposits. The images from the oligopeptide p11-4 group and Aqua demin (control) are visually indistinguishable.

Low vacuum micrographs (Figure 4) revealed superficial, net-like deposits after application of CeCl_3_. Enamel surfaces of group Oligopeptide p11-4 did not show any deposits and were not discernable from the control (Aqua demin). In group NaF and AmF, globular precipitates on the enamel surface similar to CaF_2_ deposits can be seen.

**Figure 5:**
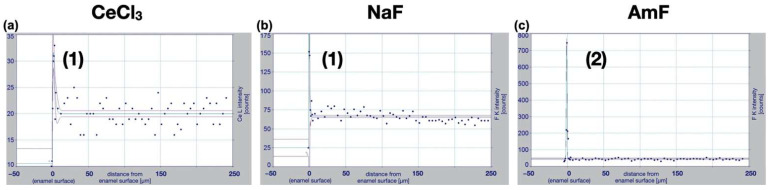
Visualization of linescans from the surface to a depth of 250 µm from the surface. For every linescan, the counts of the target element in >100 µm depth from the enamel surface resembled a horizontal line and were therefore defined as background noise. Counts (*y*-axis) of respective elements were plotted as a function of depth into enamel (*x*-axis). Two-dimensional fits were calculated (green lines) with corresponding 95% confidence limits (purple lines). Best fits were an *Equilibrium Peak function*(1) for CeCl_3_ (r^2^ = 0.62; (**a**)) and NaF (r^2^ = 0.86; (**b**)) and a *Complementary Error Peak function (2)* for AmF (r^2^ = 0.97; (**c**)).

In the cross-sectioned specimens (Figure 5), distinct surface accumulations appeared in the form of peak functions for CeCl_3_ based on the cerium counts in 3 out of 24 (12.5%) linescans, for NaF based on the fluorine counts in 20 out of 24 (83.3%) linescans, for AmF based on the fluorine counts in 23 out of 24 (95.8%) linescans, but not in oligopeptide p11-4 groups based on the nitrogen counts and not for Aqua demin (control) for any of the target elements. Notably, the peaks for CeCl_3_ and NaF are only about twice the background noise, while for AmF it is about 15 times higher.

(1) $$y=a+\frac{b}{d+e}\left[1-e^{-c\left(x-f\right)}-\frac{d}{d+e-c}\left(1+ce^{-\left(d+e\right)\left(x-f\right)}-\left(d+e\right)e^{-c\left(x-f\right)}\right)\right]$$

(2) $$y=a+b\;\mathrm{erfc}\;\left[{\left(\frac{x-c}{d}\right)}^{2}\right],\;\mathrm{with}\;\mathrm{erfc}\left(x\right)=2\;\int_{x}^{\infty }\frac{1}{\sqrt{\pi }}e^{-u^{2}}du\;;x\;\geq 0.$$

The fits showed a clear superficial accumulation of fluorine-containing deposits in all specimens in which NaF (b) or AmF (c) were applied. In some CeCl3 (a) samples, cerium-containing accumulations were detected at the surface, but with a much smaller difference between peak and background compared to the fluoride accumulations. None of the target elements Ce, N, or F were found following treatment with oligopeptide p11-4 or control (Aqua demin). A different scale (y-axis) was used for better visibility.

The elemental composition of the product containing oligopeptide p11-4 used in this study (Curodont Repair) is depicted in Table 2. The caked solution of Curodont Repair applied with the applicator brush provided by the manufacturer comprised 2.3 At%N, the solution not in contact with the respective brush 1.5 At%N, and the specific brush itself 5.6 At%N. The Curodont system contains nitrogen, indicating the presence of oligopeptide p11-4.

## 4. Discussion

In the present study, test materials with cariostatic potential were applied to sound bovine enamel. As the controls (treated with Aqua demin) always contained 0 At% of the target elements cerium (At%Ce), nitrogen (At%N), and fluorine (At%F) by median, the necessary condition to detect and analyze the cariostatic test materials used in this experimental setup was established. Moreover, the target element N proved to be reliably detectable with the EDX setup used.

For a locally applied cariostatic to actually achieve the intended preventive effects in a clinical situation, e.g., positively influencing the balance between demineralization and remineralization or inhibiting bacterial biofilm formation, it is a basic prerequisite that it can adequately attach to the enamel [5]. Despite the widespread use of fluorides, with an estimated 1.5 billion daily users of fluoridated toothpaste in 2015, dental caries is prevalent worldwide [1,19,20]. Therefore, a deeper understanding of the mechanisms of action of fluorides in the context of their cations and alternative cariostatic compounds is essential.

In a previous study, we established energy dispersive X-ray spectroscopy (EDX) as a method to detect and analyze CaF_2_-like precipitations after fluoride gel application on sound human enamel under high vacuum conditions [10]. In contrast to this former study, here we used SEM imaging and EDX, both under low vacuum conditions, to allow us to study sample surfaces without any experimental modifications, especially without a surface metal coating [21,22]. Generally, elements with larger atomic numbers (*Z* > 10) can be analyzed with high accuracy using EDX [23]. However, we were able to reliably measure elements with an atomic number ≤ 10, in the case of the present study, mainly F, O, and N, by system calibration with customized coefficient-standards (SCC) and using an EDX system with a Si_3_N_4_ window, which has higher transmittance compared to generally used polymer windows, especially for low keV X-rays emitted by elements of lower atomic numbers [24]. To date, there are no other studies directly aiming for detection of oligopeptides on dental hard tissues by targeting the nitrogen atoms using energy dispersive X-ray spectroscopy. However, measuring peptide nitrogen with EDX has already been applied in other fields such as the detection of peptides on nanofibers for peripheral nerve regeneration [25,26] or the detection of oligopeptide integrated into polymeric fibers [27].

Among the test materials applied in this study, two of the treatment solutions are marketed, clinically applicable compounds (oligopeptide p11-4: Curodont Repair; AmF: Elmex Fluid). NaF was used in the same fluoride concentration and pH-value as AmF. CeCl_3_ was prepared as an experimental solution.

At%Ce > 0 were found for all areas treated with cerium(III)-chloride without significant influence on the other elements. When applied to sound enamel, cerium might have a cariostatic effect inhibiting the onset of a carious lesion by replacing single calcium ions in the hydroxyapatite lattice, leading to a more stable substituted apatite [14,15]. Another in vitro study showed a positive effect of 10% cerium chloride application on the reduction in quantitative light-induced fluorescence loss during demineralization and remineralization cycles compared to a placebo solution [28]. Our study showed that cerium can adhere to sound enamel areas with a median of 0.8 At%Ce. Since cerium could possibly act as a substituent for calcium within the hydroxyapatite lattice, this could explain the lower Ca/P ratio of specimens treated with CeCl_3_ compared to control specimens.

After the application of the two different fluoride-containing test materials, although identical in fluoride content and pH, elemental composition differed between those two for some elements. Despite different cations, both fluoride preparations in the present study had a pH of 4, leading to precipitation of CaF_2_-like globular structures that can be seen on SEM images (Figure 4) and were confirmed by the high surface At%F. This is in accordance with other in vitro studies that showed fluoride precipitation after the application of acidic fluoride compounds [8,10,29,30]. Surface fluoride accumulation was also observed in most of the cross sectioned samples for NaF and AmF. Following application of CeCl_3_, cerium was detectable in some cross-sectioned samples, but less frequently as compared to fluorine at fluoridated samples. This can be explained by a presumably thicker layer of the precipitates containing fluoride compared to the precipitates containing cerium, which is also supported by the higher At% for the fluoride preparations compared to the At%Ce for CeCl_3_ in surface elemental composition analysis. On the other hand, two randomly located linescans were performed per sample, which is why especially thin accumulations were not detectable in all linescans, although they were revealed to be present in all surface elemental composition analyses. Oligopeptide p11-4 could neither be detected in cross sectional specimens nor by direct elemental analysis of the treated surface.

In the present study, nitrogen from oligopeptide p11-4 was found in the liquid and the applicator of the test material as depicted in Table 2, but not after application on enamel and rinsing with demineralized water, as performed for all test materials. Within the test material oligopeptide p11-4, the measurements indicate an uneven distribution of the oligopeptide over the applicator and the liquid, whereas a stable adherence of the peptide on sound enamel was not achievable. The Ca-binding capacity reported for oligopeptide p11-4 seems not to be sufficiently strong to establish its bond to the sound enamel surface.

A limitation of the study might be that we investigated the direct interaction of the applied test materials with sound polished enamel. Presence of a pellicle or pH-cycling might influence outcomes regarding superficial elemental composition. While this study shed light on the potential of novel anti-cariogenic compounds in order to prevent the onset of carious lesions starting with sound enamel, future research may focus on the persistence and efficacy of surface precipitations and in-depth penetration of such anti-cariogenic compounds to promote remineralization of previously demineralized areas varying in mineral content and porosity, in order to fully comprehend their potential during demineralization and remineralization.

## 5. Conclusions

Cerium and fluorine could be detected significantly on all bovine enamel surfaces after application of CeCl_3_ and NaF or AmF, respectively, which were found to be significantly different to the untreated control. In contrast, nitrogen was not detected after application of oligopeptide p11-4 and did not lead to any significant difference in superficial elemental composition compared to untreated control specimens.

## Figures and Tables

**Table 1:** Results of atomic percent [At%] and Ca/P ratio at the enamel surface following treatment according to group (n = 12). Since data are not symmetrically distributed, and some of the sums of the medians may differ from 100%. Asterisks indicate significant difference between the respective group and the control (Aqua demin). Data are also depicted in Figure 2 and Figure 3.

Group										
	Aqua Demin (Control)		CeCl_3_		Oligopeptide p11-4		NaF		AmF	
	Median	25–75%	Median	25–75%	Median	25–75%	Median	25–75%	Median	25–75%
**At%Ce**	0.00	0.00–0.00	0.75 *	0.60–1.43	0.00	0.00–0.00	0.00	0.00–0.00	0.00	0.00–0.00
**At%N**	0.00	0.00–0.00	0.00	0.00–0.00	0.00	0.00–0.00	0.00	0.00–0.00	0.00	0.00–0.00
**At%F**	0.00	0.00–0.00	0.00	0.00–0.00	0.00	0.00–0.10	15.20 *	12.88–16.85	17.04 *	8.85–21.23
**At%Ca**	21.85	20.35–22.63	20.90	19.85–22.08	20.80	20.25–22.08	22.35	21.53–23.15	24.55 *	23.58–25.48
**At%P**	13.40	12.63–13.77	13.20	12.85–13.60	13.00	12.58–13.51	11.00 *	10.43–11.18	11.22 *	10.38–12.90
**At%O**	63.60	62.62–65.78	64.10	62.50–64.98	65.00	63.19–66.18	50.70 *	48.93–52.18	47.55 *	40.41–52.38
**At%Na**	0.40	0.40–0.50	0.40	0.40–0.40	0.40	0.40–0.50	0.60 *	0.53–0.60	0.45	0.40–0.50
**At%Mg**	0.50	0.40–0.60	0.43	0.40–0.58	0.40	0.40–0.50	0.60	0.43–0.70	0.50	0.40–0.64
**Ca/P ratio**	1.63	1.62–1.65	1.60 *	1.55–1.62	1.62	1.60–1.63	2.03 *	1.96–2.12	2.17 *	1.92–2.40

**Table 2:** Relative elemental composition of the manufacturer’s applicator brush, the test material Oligopeptide p11-4 applied according to manufacturer’s instructions on a Leit-Tab, the oligopeptide p11-4 fluid on a Leit-Tab, and the Leit-Tab without test material application.

Element [Atom%]	C	N	O	Na	S
Oligopeptide p11-4 manufacturer’s applicator brush	63.4	5.6	31	0	0
Oligopeptide p11-4 applied on Leit-Tab according to manufacturer	54.3	2.3	43.3	0	0.1
Oligopeptide p11-4 fluid on Leit-Tab	66.1	1.5	30.9	0.7	0.8
Leit-Tab without test material application	87.5	0	12.1	0.1	0.3

## Data Availability

All relevant data generated or analyzed during this study are included in this article. Further enquiries can be directed to the corresponding author.
